# Understanding the Views of Health Care Professionals on the Usability and Utility of Virtual Reality Multidisciplinary Team Meetings: Usability and Utility Study

**DOI:** 10.2196/60651

**Published:** 2025-02-14

**Authors:** Maryam Almashmoum, Antony Payton, Emily Johnstone, James Cunningham, John Ainsworth

**Affiliations:** 1 Division of Informatics Imaging and Data Sciences, School of Health Sciences, Faculty of Biology, Medicine, and Health, University of Manchester, Vaughan House, Portsmouth St., Manchester, M13 9GB, United Kingdom, 44 07949062010; 2 Nuclear Medicine Department, Faisal Sultan Bin Eissa, Kuwait Cancer Control Center, Kuwait City, Kuwait; 3 VREvo Ltd, The University of Manchester Core Technology Facility, Manchester, United Kingdom; 4 NIHR Manchester Biomedical Research Centre, Manchester University Hospitals NHS Foundation Trust, Manchester Academic Health Science Centre, Manchester, United Kingdom

**Keywords:** knowledge sharing, multidisciplinary team meetings, artificial intelligence, heuristic evaluation, usability, virtual reality, VR, simulation, virtual environments, digital environments

## Abstract

**Background:**

Multidisciplinary team (MDT) meetings are one of the facilitators that enhance knowledge sharing among health care professionals. However, organizing a face-to-face MDT meeting to discuss patient treatment plans can be time-consuming. Virtual reality software is widely used in health care nowadays to save time and protect lives. Therefore, the use of virtual reality multidisciplinary team (VRMDT) meeting software may help enhance knowledge sharing between health care professionals and make meetings more efficient.

**Objective:**

The objectives of this study were to introduce VRMDT software for enhancing knowledge sharing and to evaluate the feasibility and usability of the VRMDT for use by professionals in health care institutions.

**Methods:**

We invited participants from The University of Manchester Faculty for Biology, Medicine, and Health who had a health care background. As this was the first stage of software development, individuals who did not usually attend MDT meetings were also invited via email to participate in this study. Participants evaluated VRMDT using a Meta Quest 3 headset, and software developed using the Unity platform. The software contained an onboarding tutorial that taught the participants how to select items, load and rotate 3D Digital Imaging and Communications in Medicine files, talk to a generative artificial intelligence–supported avatar, and make notes. After the evaluation (approximately 15 min), participants received an electronic survey using the Qualtrics survey tool (Qualtrics International Inc) to score the usability and feasibility of the software by responding to the 10-item system usability scale, and 12-point heuristic evaluation questions with Neilsen severity rating.

**Results:**

A total of 12 participants, including 4 health informatics, 3 with a nursing background, 2 medical doctors, 1 radiologist, and 2 biostatisticians, participated in the study. The most common age bracket of participants was 20‐30 years (6/12, 50%). Most of the respondents had no experience with virtual reality, either in educational or entertainment settings. The VRMDT received a mean usability score of 72.7 (range between 68 and 80.3), earning an overall “good” rating grade. The mean score of single items in the heuristic evaluation questionnaires was less than 1 out of 4 (the overall mean was 0.6), which indicates that only minor problems were encountered when using this software. Overall, the participant’s feedback was good with highlighted issues including a poor internet connection and the quality of the generative artificial intelligence response.

**Conclusions:**

VRMDT software (developed by Sentira^XR^) was developed with several functions aimed at helping health care professionals to discuss medical conditions efficiently. Participants found that the VRMDT is a powerful, and useful tool for enhancing knowledge sharing among professionals who are involved in MDT meetings due to its functionality and multiuser interactive environments. Additionally, there may be the possibility of using it to train junior professionals to interpret medical reports.

## Introduction

### Overview

The United Kingdom’s health care sector is facing significant pressures from increased patient demands and workforce supply issues. A need for efficiently connected health care employees is important for sharing knowledge and it is an integral part of knowledge management. During COVID-19, communication across sectors moved towards web-based communication methods [1-3], such as videoconferencing (eg, Microsoft Teams and Zoom), which helped to protect the lives of patients and staff [3-5]. To maintain knowledge-sharing practices among professionals, there are several professional digital communities [6,7]. The purpose of these professional digital communities is to get professionals with common expertise to share their knowledge without considering geographical barriers [6]. Virtual multidisciplinary team (MDT) meetings have been shown to have a visible role in maintaining communication among cancer care professionals to discuss, follow up, and set clear treatment plans [8]. Additionally, it has been shown to improve cancer patients’ outcomes [9-13]. Traditional face-to-face methods of MDT have drawbacks that limit attendance including lack of time and funding [8]. Introducing new technology for communication has benefits, although there are also drawbacks such as reliance on bandwidth, increased conversation time, and loss of gesture communication that can be difficult compared with traditional methods, thereby directly affecting good decision-making [14,15].

The use of videoconferencing has surged as a communication method during and post-COVID, although it has limitations including the inability for natural F-2-F interaction due to the participants only seeing a video image. Additionally, smooth and stable internet network is required to ensure that video conferencing runs smoothly. Moreover, the inability to show 3D images compared with the virtual reality (VR) tools may be a distinct disadvantage [16]. As a result, the existence of a powerful web-based tool that simulates a real environment may have benefits. VR and augmented reality are increasingly being used in the medical field both for training and as a procedural aid [17]. VR is defined as “a three-dimensional computer-generated simulated environment, which attempts to replicate real world or imaginary environments and interactions, thereby supporting work, education, recreation, and health” [3,18]. In addition, the user can interact with avatars using generative artificial intelligence (AI) supported natural language processing (NLP) which further enhances the realism of the experience. It requires head-mounted displays, and either hand controllers or hand tracking in order to perform practical procedures [19]. The sense of presence is one of the key characteristics of VR that makes it different from other communication mediums [14]. The use of VR applications in the health care market has grown massively in recent years. In 2022, the VR health care market reached over US $2.3 billion worldwide, with 171 million VR users [20].

VR in health care has several benefits, such as facilitating training, education, and the development of technical skills. Additionally, VR is being used for a variety of purposes, including surgery and treatment, training, and patient therapy and rehabilitation [21]. Kyaw et al [22], illustrated that using VR applications improves professionals’ skills, and knowledge compared with face-to-face communication and web-based digital education. In particular, it has the ability to negate the need for face-to-face contact, while maintaining the illusion of being with colleagues in the real world [23].

There are several factors that affect knowledge sharing in the medical imaging department at cancer centers, which are similar to those in most health care sectors [24]. MDTs are considered important departmental facilitators that enhance knowledge sharing among health care professionals [24]. MDT is considered a pillar of the best practices in cancer canters and plays an important role in cancer Treatment [25]. The United Kingdom’s National Health Service definition of MDT is “a group of professionals from one or more clinical disciplines who together make decisions regarding the recommended treatment of individual patients” [26]. MDT in cancer centers is defined as the collaboration of several health care professionals in different fields engaged in the treatment of cancer with the overall objective of enhancing the rate of interpreting treatments of cancer patients, and patient care [13,26]. Cancer centers began to use a multidisciplinary approach in the mid-1980s, and by the 1990s, the MDT meeting was introduced as an instrument for providing coordinated, collaborative care, which allow a broader range of opinions on treatment plans [13,27]. In addition, it provides training for junior health care professionals. However, there are several barriers that contribute to not attending those meetings as per policy recommendations. These include time constraints, lack of departmental arrangements, geographical barriers among health care professionals, and shortage of staff [13].

In health care institutions, implementing new interventions such as VR among health care professionals may overcome current barriers and enhance knowledge-sharing practices to increase patients’ outcomes and minimize medical mistakes. However, there are several challenges to implementing VR as a communication tool, including providing evidence that these technologies can save time, increase productivity, and reduce carbon footprint, without adding significant hardware costs and training time [28-30]. The aim of this research is to introduce new technology and perform a usability study of VR in MDT to investigate the feasibility and usability of using VR in cancer health care meetings.

### Objectives

In this study, we developed a virtual reality multidisciplinary team (VRMDT) for enhancing communication with professionals, which was evaluated in terms of its usability by professionals from a variety of backgrounds.

The aim of this study was to investigate the usability of newly developed VRMDT software that helps gather health professionals in a 3D immersive environment to aid communication and set a clear treatment plan for the cancer patient. The objectives of this study were:

- To introduce VRMDT software to health care professionals.
- Evaluate the usability, feasibility, and efficacy of VRMDT by applying the System Usability Scale (SUS), and identifying the problems with the user interface by using a heuristic evaluation questionnaire.
- Identify the strengths and weaknesses of using VRMDT.
- Determine if this technology has the potential to increase the number of MDT meetings in cancer centers locally and internationally.
- Increase awareness of using VR technology among health care professionals in cancer centers.

## Methods

### An Overview of VRMDT Software

The software was designed by our University of Manchester research team and developed using the Unity platform by Sentira^XR^ [31], which is a University of Manchester spinout that uses VR and generative AI NLP to create authentic training simulations for health care professionals and other disciplines. The designs of the VRMDT comprise:

- An onboarding section for those not familiar with VR.
- Options to select a health care uniform of varying color and add the name to be displayed above the head of each user’s avatar.
- 3D VR meeting room with round table.
- Ability to display a 3D Digital Imaging and Communications in Medicine (DICOM) scan image in the middle of the virtual table to allow 3D visualization. Additionally, there is a screen in front of each user to few the DICOM images in a traditional 2D mode.
- A whiteboard for writing notes and drawing images.
- A laser pointer beside each user for pointing to specific locations on the 3D DICOM images.
- An interactive avatar that uses generative AI NLP to provide answers to questions from users in the room related to the patient’s scans, condition, and patient history.
- A master control panel where patient DICOM images can be selected.

**Figure 1.**
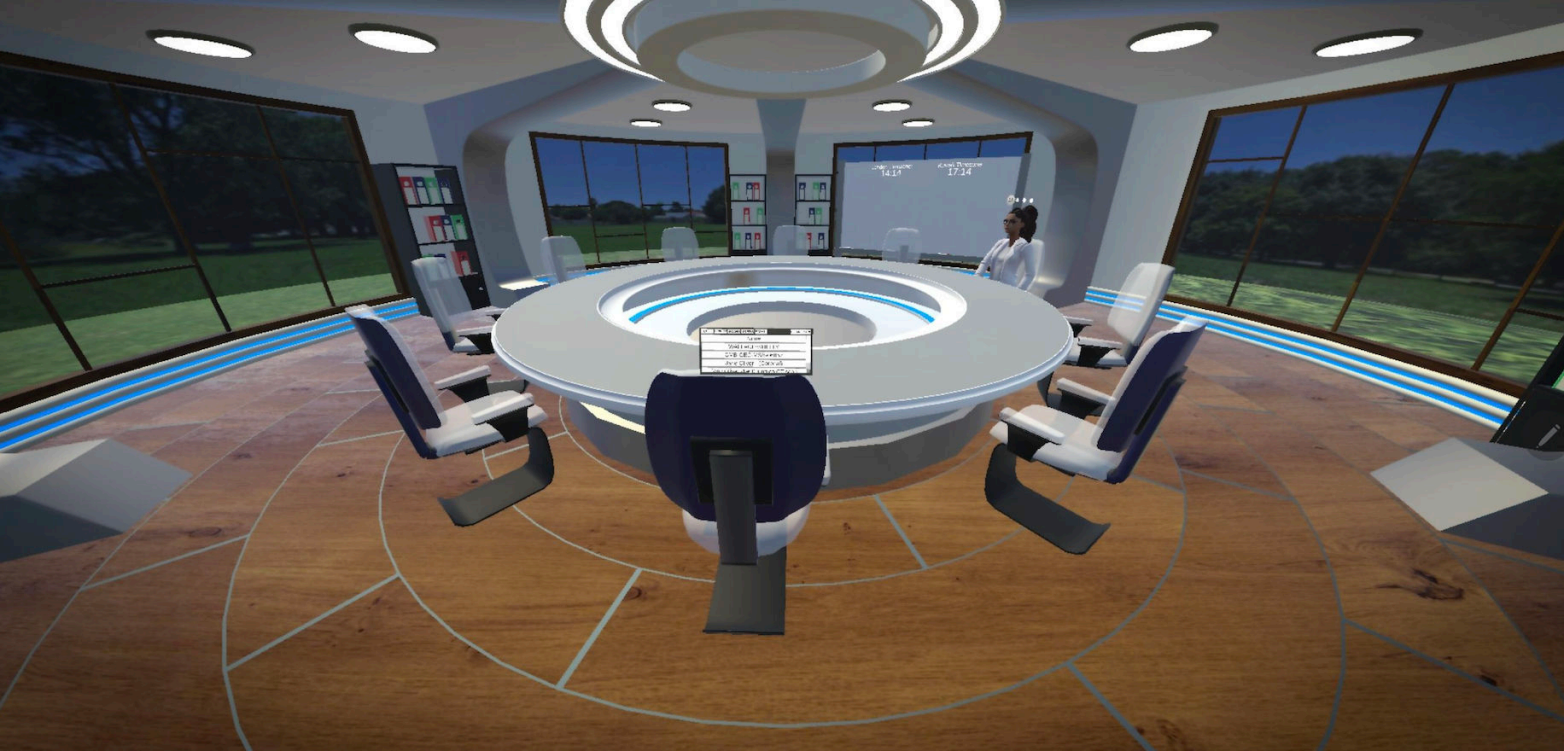
The environment of the virtual reality multidisciplinary team software.

**Figure 2.**
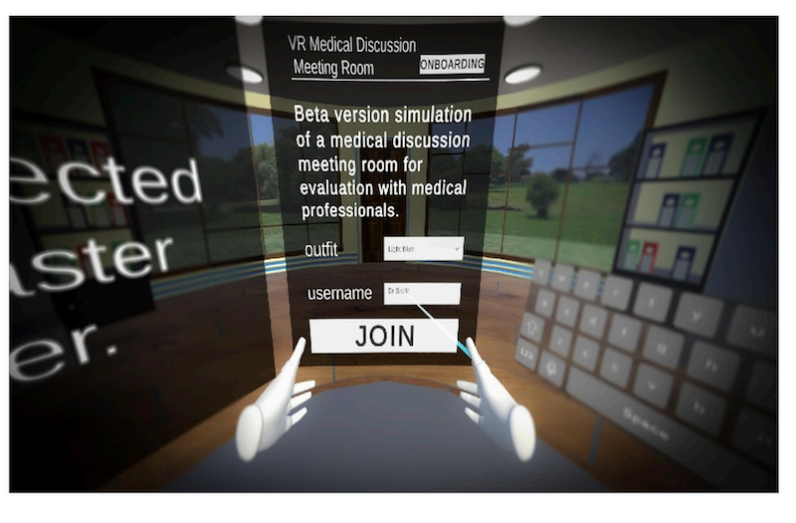
“On boarding” interface page for selection of the outfits, and the info that will appear on the user (such as name).

**Figure 3.**
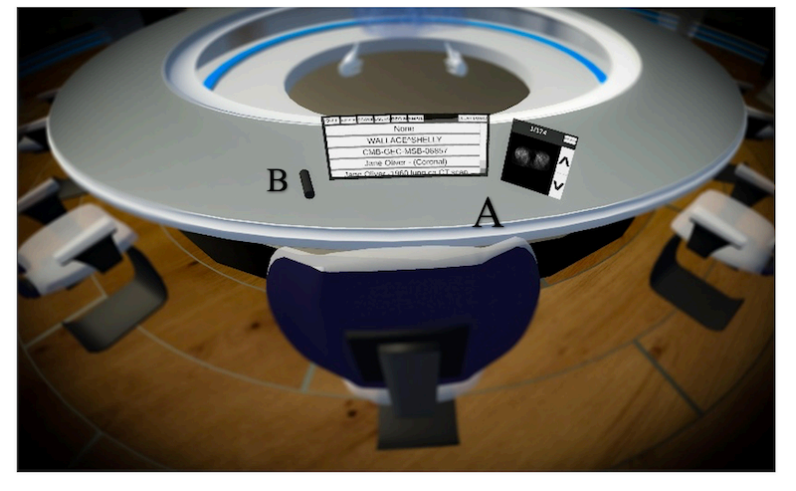
(**A**) Two screens: a controlled screen and a screen to display the traditional 2D scan images. (**B**) Laser pointer.

**Figure 4.**
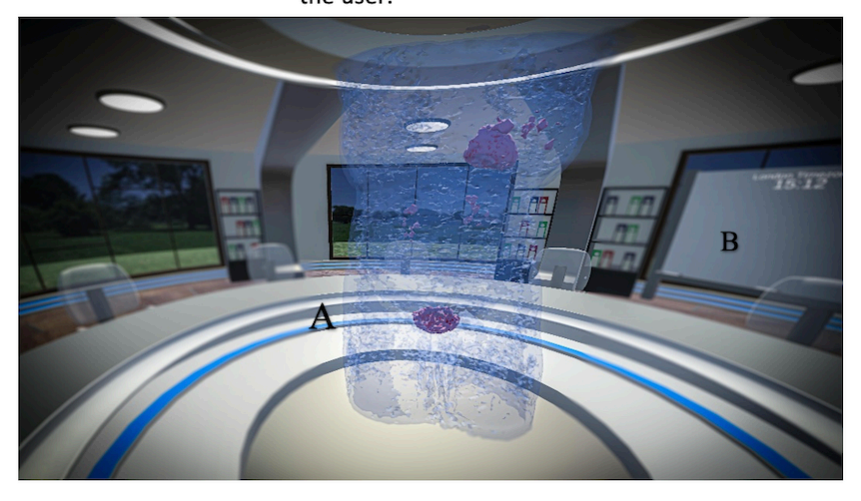
(**A**) The 3D Digital Imaging and Communications in Medicine (DICOM) images and (**B**) a whiteboard.

The VRMDT (Figure 1) is designed to allow health care professionals to treatment plan anywhere and at any time. To run the VRMDT simulation, a reasonable Wi-Fi connection (≥10 Mbps), head-mounted display, and controllers are required. Before entering the MDT room, the user had the option to undertake an onboarding scenario that introduced them to basic functionality such as picking up objects, talking to the avatar, selecting DICOM files, and making notes on a whiteboard. The user can then begin the simulation first by typing in their username (displayed over the head of their avatar) and selecting their outfit’s color (Figure 2). In the VRMDT software, there is a round table fitting 10 users with a control screen that contains the setting options, selecting the patient DICOM files, and the option to move the control panel to another user. Another screen available to all 10 users displays the traditional 2D DICOM images for cancer patients (Figure 3B). Additionally, the meeting room contains a whiteboard to allow the user to make notes or draw diagrams (Figure 4B). In the middle of the meeting table, the 3D DICOM (Figure 4A) images appear with the facilities to rotate the images on the x-axis to help show any tumors or lesions. A laser pointer is available to each participant to help highlight a region on the 3D image (Figure 3A). DICOM images were retrieved from The Cancer Imaging Archive which are accessible for the public to download and use without ethical approval. The time zones for both the United Kingdom and Kuwait are displayed on the wall of the meeting room.

**Figure 5.**
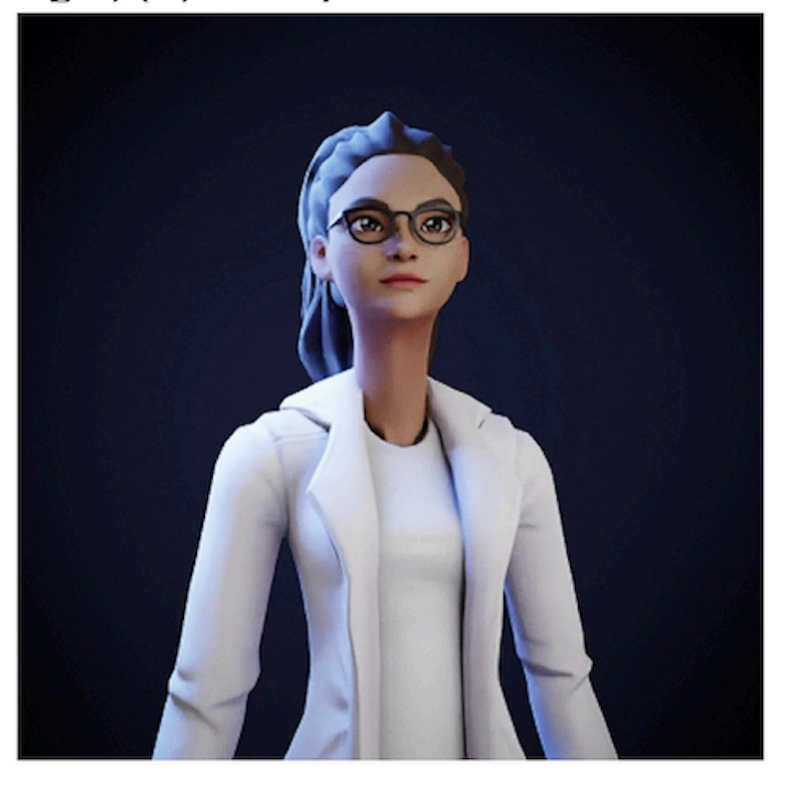
Interactive avatar.

Generative AI NLP used the InWorld platform [32]. Voice cloning (voice of MA cloned) uses Eleven Labs software which is supported by InWorld [32,33]. Patient information and avatar background details were entered into InWorld and quality assurance was conducted to ensure that the responses from the generative AI NLP had an accuracy of 95% or greater. The generative AI NLP-supported avatar was placed in the meeting room (Figure 5) and allowed the user to ask questions regarding the medical condition of the patients. The Photon platform was used to allow users to speak with each other as they would with any teleconference software [34]. The purpose of the AI-supported avatar was to provide the MDT with specific details on each of the patients, such as name, age, status of the medical condition, medications, chemotherapy/radiotherapy received, response to treatments/medications, bloodwork, and patient concerns. Providing patient information via an avatar, removed the need for reading extensive text notes which is not ideal in a VR environment due to reduced visual resolution and an increased risk of cybersickness. It also allowed for one or more of the MDT to be absent and still provide the information.

For the implantation, the software required a direct connection with the Picture and Archiving and Communication System to visualize patient images. Additionally, the VRMDT contains instructions voiced over to guide the user throughout testing the software.

### Participants

To be eligible for participation in this study, the participant had to have a health care background, with those recruited being postgraduate students and staff at The University of Manchester. As this was the first stage of software development, participants who were not routinely involved in MDTs were also invited to evaluate the software.

Participants were recruited via email with the inclusion criteria as provided in Textbox 1.

Textbox 1. **Inclusion and exclusion criteria.**

Inclusion criteria

- Postgraduate students and staff at the University of Manchester.
- 21 years or older.
- Any gender.
- Health care professional background (including but not limited to doctors, nurses, and radiologists).
- Health care professionals who are involved in multidisciplinary teams.
- Willing to provide informed consent.
- English speakers.
- No pre-existing conditions that may cause discomfort or distress in a virtual reality (VR) environment.

Exclusion criteria

- People who do not read, speak or understand English, because the software is in English only.
- People who are unwilling to wear a VR headset.
- People who had a pre-existing condition that may cause discomfort or distress in a VR environment

### Instruments

Validated usability and utility questionnaires were used to assess the simulation’s efficacy, efficiency, and user pleasure [35]. Two methods were used to assess the usability evaluation: 10-item SUS, and 12-item heuristic evaluation questionnaires [36,37]. Upon completion of the trial, the SUS and heuristic questionnaire links were emailed to the participant to complete in their own time in Multimedia Appendix 1. The survey was built using the Qualtrics survey tool [38]. Participants were asked to assess the software based on 10-point scales [36] and answer statements using a 5-point Likert [36,39-46]. The SUS was selected as it is suitable method when applied to a small sample size (N less than 14) [39]. Questions 1, 3, 5, 7, and 9 are positive, whereas questions 2, 4, 6, 8, and 10 are negative. The 10 connected questions provide a full review of a product. The SUS yields a score between 0 and 100 [47]. A higher SUS score is associated with greater product usability.

To evaluate the user interface, and identify problems with the software, heuristic evaluation was used [37]. There are several heuristic evaluation questionnaires used to assess human-computer interaction [37,48,49]. In this study, we used the heuristic evaluation questionnaire based on Sutcliffe and Gault’s heuristic evaluation of VR apps [37]. It consists of 12 heuristic items, including natural engagement, compatibility with the user’s tasks and domain, natural expression of action, close coordination of actions and representation, realistic feedback, faithful viewpoints, navigation and orientation support, clear entry and exit points, consistent departures, support for learning, clear turn-taking, and sense of presence.

Our survey was an open survey (no password required) based on several previous VR usability studies but modified slightly to align with our simulation [36,37]. The survey was checked by 10 individuals with a health care background to ensure it was easy to understand. In addition to the SUS questions and heuristic evaluations, we also collected information on demographics.

### Procedure

At the beginning of the evaluation, participants were given a brief introduction to the project and shown how to use the VR headset and controllers. For those new to VR, an onboarding section was available. The overall evaluation ran for approximately 10 to 15 minutes. If there was more than 1 participant present at the same time, we allowed them to trail the software together so that they could see and interact with each other through the VRMDT. For those who evaluated solo, one of the development team would join them in the simulation so they could experience multiuser functionality. The participants were emailed the survey to complete within a 2 week time frame with a reminder sent after this period. Evaluations were conducted between February and March 2024). All sessions are located at The University of Manchester in a dedicated VR lab.

### Data Interpretation

The results are interpreted as a grade for the SUS and a mean for the heuristic evaluation. To provide the grade of the SUS, there are 4 ratings for SUS interoperation: excellent (score greater than 80), good (69‐80.3), okay (score equal to 68), poor (51-68), and awful (less than 51) [36]. For the heuristic evaluation, each item was rated for severity using Nielsen scale (no problem=0, cosmetic problem=1, minor problem=2, major problem=3, and catastrophe=4), as shown in Table 1 [47]. Only completed questionnaires were included in the final results.

**Table 1.** Nielson severity rating [48].

Rating	Definition
Don’t Agree	I do not agree that this is a usability problem at all (there are no problems with usability)
Cosmetic problem	Needs not to be fixed unless extra time is available on the project (if there is time, aesthetic issue that only has to be fixed).
Minor problem	Fixing this should be given low priority (a low priority for a minor usability problem).
Major problem	Important fix required that should be given high priority (major usability problems, must be fixed right away)
Catastrophic	Imperative to fix this before product can be released.

### Data Analysis

The final data were analyzed by entraining it into an Excel spreadsheet where the SUS score was calculated and the rate of the severity of each heuristic item based on the Nielsen severity scale for each item. The SUS questionnaire consisted of 10 questions. The score of SUS was calculated by adding the odd questions minus 5 and 25 minus the even number then multiplied by 2.5 [36]. On the other hand, the rate of heuristic severity was calculated by adding the number of statements and accepting the first statement which is no problem because it has zero value [37].

### Ethical Considerations

The main purpose of this study is an anonymized evaluation of the VRMDT software in terms of its usability and utility. Therefore, the University of Manchester web-based ethics tool and the School of Health Sciences ethics representative confirmed that ethical approval was not required for this study. Consent was obtained from all participants that required them to sign a consent form. Anonymized responses were securely saved using the Qualtrics database.

## Results

### Participants

A total of 12 participants from a variety of health care fields were recruited (8/12, 67% female; 4/12, 33% male) with half of the participants being between 20 and 30 years of age (6/12, 50%). Most of the volunteers had a doctorate degree (8/12, 67%), with 4 having experience in health informatics. Most of the participants had no experience using VR before the evaluation. The demographic characteristics of the respondents are shown in Table 2.

**Table 2.** Demographics characteristics of the respondents (N=12).

Characteristics	Values, n (%)
Sex	
Female	8 (67)
Male	4 (33)
Age group (years)	
20-30	6 (50)
30-40	5 (42)
50-60	1 (8)
Highest education level	
Master degree	8 (67)
Doctorate degree	4 (33)
Background	
Nursing	3 (25)
Radiologist	1 (8)
Health Informatics	4 (33)
Medicine	2 (17)
Biostatistics	2 (17)

### Usability (SUS Questionnaires)

A total of 67% (n=8) of participants gave SUS scores greater than or equal to 68. Four (33%) of the participants scored “Poor” with the VRMDT, with the SUS score rate less than 62. The total mean score was 72.7, resulting in an overall “Good” rating. The SUS scores for the respondents are shown in Table 3.

Multimedia Appendix 2 presents the interpretation of the SUS. Based on the SUS items, the participants indicated that the software was easy to learn how to use, with a mean score of 4.1. The highest score was given to the item “I found the various functions in this software were well integrated (eg, whiteboard, and DICOM images)” with a mean score of 4.25. In contrast, the lowest score was given to the item “I thought there was too much inconsistency in this software” with a mean score of 1.5 where low scores are an indicator of better consistency.

**Table 3.** System Usability Scale (SUS) scores for respondents. Average=72.7 (Good).

Respondents	Results			
X0^a^	Y0^b^	SUS	Grade^c^
1	19	15	85	A
2	17	17	85	A
3	17	12	72.5	B
4	12	19	77.5	B
5	16	9	62.5	D
6	7	14	52.5	D
7	16	19	87.5	A
8	14	14	70	B
9	14	9	57.5	D
10	11	10	52.5	D
11	18	16	85	A
12	18	16	85	A

a The total odd SUS questions–5.

b 25–the total even SUS questions.

c Sum of X0 and Y0 × 2.5 (A=Excellent, B=Good, C=Okay, D=Poor, and F=Awful).

### Heuristic Evaluation

The participants rated the severity of each heuristic item based on Nielsen severity scale. The results of these ratings are shown in Table 4. The value of the first severity scale “no problem” is zero, so it was not counted. We estimated the number and severity of reported problems for each item. For example, we received 3 statements that indicated the minor problems for the first item “natural engagement,” 1 for the major problem, and 2 for the cosmetic problem. The total score was calculated by adding each heuristic item. All the items had a usability score of less than 12, with a mean score of less than 2. This indicated well-functioning software.

The summary rate is shown in Table 5. One of the respondents reported 32 problems and 3 indicated no problems at all based on 12 heuristic items.

**Table 4.** Heuristics evaluation for each item with Nielson severity rating.

Number of items of the heuristics	Nielsen severity rating						
	No problem (0)	Cosmetic problem (1)	Minor problem (2)	Major problem (3)	Catastrophe (4)	Total	Mean
1. Natural engagement	9	0	2	1	0	3	0.7
2. Compatibility with the user’s task	6	2	2	2	0	6	1.5
3. Natural expression of action	6	2	3	0	1	6	1.5
4. Close coordination	8	2	0	1	1	4	1
5. Realistic feedback	87	2	1	2	0	5	1.2
6. Faithful viewpoint	10	0	2	0	0	2	0.5
7. Navigation and orientation support	10	1	0	1	0	2	0.5
8. Clear entry and exit point	9	1	1	1	0	3	0.7
9. Consistent departures	8	3	1	0	0	4	1
10. Support for learning	7	0	4	1	0	5	1.2
11. Clear turn	11	0	0	1	0	1	0.2
12. Sense of presence	8	1	2	1	0	4	1

**Table 5.** Heuristics evaluation with Nielson severity rating for each respondent (resp).

Number of items of the heuristics	Respondents scores												
	Resp.1	Resp.2	Resp.3	Resp.4	Resp.5	Resp.6	Resp.7	Resp.8	Resp.9	Resp.10	Resp.11	Resp.12	Total
1. Natural engagement	0	0	0	2	3	0	0	2	0	0	0	0	0.5
2. Compatibility with the user’s task	0	2	0	0	2	3	0	3	0	1	1	0	1
3. Natural expression of action	0	1	0	0	4	2	0	2	0	2	1	0	0.7
4. Close coordination	1	0	0	0	4	0	1	3	0	0	0	0	0.4
5. Realistic feedback	0	0	0	2	3	0	1	3	0	0	1	0	0.8
6. Faithful viewpoint	2	0	0	0	0	0	0	2	0	0	0	0	0.3
7. Navigation and orientation support	0	0	0	1	0	0	0	3	0	0	0	0	0.3
8. Clear entry and exit point.	0	0	0	2	0	0	1	3	0	0	0	0	0.5
9. Consistent departures	0	1	0	1	0	0	0	2	0	1	0	0	0.4
10. Support for learning	2	0	0	2	0	0	0	3	0	2	2	0	1
11. Clear turn	0	0	0	0	0	0	0	3	0	0	0	0	0.2
12. Sense of presence	0	0	0	2	0	2	0	3	0	0	1	0	0.7
Total	5	4	0	12	8	7	3	32	0	6	6	0	0.6

## Discussion

### Principal Findings

The findings of this study provide valuable insight into the current usability and future improvements of VRMDT software. Previous research into VR meeting rooms indicates that they may be an efficient tool for improving communication during the planning of patient treatments [50]. Kirchgessner et al [51] illustrated that VR meeting rooms are more motivational than traditional technologies such as Zoom. Our work supported this with participant comments mentioning that presenting DICOM images in both 2D and 3D formats made the VR meeting more efficient than standard videoconferences with, respondent (D) mentioning “Being able to view images in 3D is the best thing about the VR software.”

Our results found that the VRMDT software had adequate usability, with a mean SUS of 72.7, which is classed as “Good” as an overall interoperation. Most of the participants indicated that the simulation does not require intensive training to use it, suggesting that the inbuilt onboarding software is sufficient for training purposes, the respondent (C) said that “Browsing menus was simple and they were easy to use. Viewing DICOM images was intuitive.” This is important for any health care institution as it will reduce the impact on existing training budgets and trainer time. Additionally, most of the respondents indicated that the software contains several useful functions, such as 2D and 3D DICOM views, a whiteboard, and an avatar that responds naturally to questions. These results suggest our software has clear advantages compared with conventional teleconferences. Another positive feedback was that the immersive 3D meeting room environment helped users feel as though they were in a real-world meeting. It is worth mentioning that a low score (mean=1.5) was given to the item “I thought there was too much inconsistency in this software,” which indicated that the software was more relevant to its aim and objectives, and it performed well. The heuristic evaluation method indicated that the VRMDT has a good user interface with a low number of reported issues.

### User Experience

Participant feedback highlighted a few areas for improvement. Respondent (A) illustrated that “The reason why I indicated there were some problems was due to the internet connection not being stable, which sometimes led to lagging and the AI avatar being slow to respond,” and another respondent (B) said that “Software has potential but requires good Wi-Fi connection.” Therefore, one of the major issues indicated by most of the users was the poor internet connection, which effected the sense of presence and interaction with some functionality. Additionally, the internet connection effected the interaction with the avatar which resulted in delayed responses to questions. This was an issue with the evaluation room which received a poor internet signal and was not an issue with the software. The other issue was related to the avatar. The respondents mentioned that the AI needed to be further developed to respond to more specific clinical questions other than age, general treatment, and health conditions. Additionally, it should be designed to respond to any questions with different accent words, the respondent (C) said that “It also struggled with my accent for certain words.”

On the other hand, most of the respondents indicated that VRMDT was a powerful tool for sharing knowledge digitally compared with the other mediums because it contains several functions that make the environment immersive and very close to reality. Respondent (A) said that “it felt very futuristic, and I feel it will play an important role in future trans-geographical meetings.” Therefore, this software would be a good alternative tool in the future when face-to-face communication is not possible. Additionally, it was suggested that VRMDT may be an alternative tool for training and assessing the knowledge of junior professionals instead of in-person training. In the future, I would like to update the software by adding several functions that help in upgrading the current software. For instance, the meeting room will be secure under each hospital’s policies. In addition, those who have permission to enter this room can join this meeting after the invitation occurs. Moreover, It will contain the digital library, which contains the files and information about the cases that you want to make decisions regarding those cases.

Overall, the simulation was identified as a powerful tool for VR clinical meetings. In particular, it contained a functionality that allowed users to view both 3D and 2D DICOM images. While this has also been developed for off-the-shelf software (eg, [52]), the other software does not cater to a larger number of users generally seen at clinical meetings and lacks additional functionality such as a whiteboard, laser pointer, and AI-assisted avatar. Indeed, the avatar as an AI assistant was generally found to be very helpful in answering questions regarding the patients’ condition and was found to elevate the usability of the VR meeting. Previous independent work has suggested that cybersickness is an issue for some users [29,30,53]. That issue was not indicated in the user’s feedback from our study. The reasons for cybersickness not being an issue may include that the simulation was developed so the user can remain seated, which reduces excessive body movement both in real life and the simulation and provides a comfortable body position. Second, the headsets were modern (Meta Quest 3’s with battery strap) and had a high frame rate (90 Hz), with a wide field of view (110°H × 96°V), which also helps reduce the risk of sickness. The Quest 3 headset is also reasonably priced (£480; US $596) and easy to set up and use, making it a cost-scalable solution. We also found that the software was usable in the Meta Quest 2 without significant loss in performance, with this headset being a much cheaper option (£200; US $249.45). Overall, the hardware experience was good, with users finding the headset very light on their head, and the controllers easy to use. As a first-time exposure to VR, the majority found the experience “amazing” enough that they recommended its implementation for future VR meetings.

### Limitation and Future Studies

This study has several limitations that are worth documenting, and which we will consider for future developments. First, the VRMDT software was evaluated by a small number of health care professionals. Second, most of the volunteers were researchers, and many were from the health informatics field. Third, we encountered another issue that the evaluation took place in a room that had a poor internet connection. That limited the testing of the software efficiently, particularly the avatar generative AI NLP which had lag, and multiuser functionality where verbal communication between users was slightly delayed. Finally, the generative AI seemed limited in answering questions related to the patient’s condition due to the lack of information available on the archival system.

Future research will need to consider testing using a more statistically powerful number of health care professionals involved in MDT meetings to determine how powerful the 2D or 3D DICOM images are at identifying cancer lesions. Second, to overcome the internet issue, we need to test the network stability before performing the usability study. Thirdly, the AI generative avatar needs to be supplied with more detailed knowledge about the patients so it can more accurately answer. Additionally, a longitudinal analysis after implementation would allow researchers to assess the impact of the software on productivity. Finally, a direct comparison of our software with current digital tools such as Zoom and Microsoft Teams will help to assess its usefulness in terms of features, and productivity.

### Conclusions

In health care institutions, applying knowledge management is crucial to using resources in a good way to increase patients’ outcomes, and reduce medical errors. Knowledge sharing is considered an important step for the successful implementation of knowledge management. There are several factors that affect knowledge sharing in medical imaging. These factors can be divided into 3 categories: individual, departmental, and technological factors. MDT meetings are considered a crucial departmental factor in enhancing knowledge sharing. However, time constraints and geographical barriers can impact knowledge exchange efficiency. We have shown that creating a VRMDT meeting room may be a powerful tool to reduce those barriers.

Our VRMDT allowed the volunteers to interact with other users, and use the specialized features that allowed them to understand the patient’s condition and scans in a correct and efficient way with the volunteers rating the simulation as good. Our results suggest that multiuser VR meeting rooms that use generative AI, and the ability to visualize DICOM files in both 2D and 3D have advantages over currently used meeting methods and would benefit from further development and research.

Future development and research by our group would evaluate the usability with a wider range of health care staff and an increased number of volunteers, and overcome the limitations that were outlined in this study. We also intend to explore software security for connecting to health care systems in order to access patient scans and data and develop the software across platforms to include a wider range of VR headsets as well as PCs and tablets.

## Supplementary material

10.2196/60651 Multimedia Appendix 1 The consent form and the questionnaire of the survey.

10.2196/60651 Multimedia Appendix 2 The interpretation of the System Usability Scale and heuristic evaluation.

10.2196/60651 Checklist 1 STROBE Checklist.

## Abbreviations

AI: artificial intelligence
DICOM: Digital Imaging and Communications in Medicine
MDT: multidisciplinary team
NLP: natural language processing
SUS: System Usability Scale
VR: virtual reality
VRMDT: virtual reality multidisciplinary team
