# Supplementary material for: Understanding the Views of Health Care Professionals on the Usability and Utility of Virtual Reality Multidisciplinary Team Meetings: Usability and Utility Study
Source: JMIR XR Spat Comput. 2025 Feb 14;2:e60651. doi: 10.2196/60651 (PMC12671323; doi:10.2196/60651)
Supplement: Multimedia Appendix 1 [file xr-v2-e60651-s001.docx]

**Understanding the views of health care professional on the usability and utility of virtual reality multidisciplinary team meetings VR(MDT)**

**Dear participants,**

             Thank you for taking time to complete this questionnaire to assist with my PhD research. I am currently undertaking a Ph.D., at the University of Manchester, exploring knowledge sharing in medical imaging departments.
In my PhD We have developed a Virtual Reality Multidisciplinary Team meeting software (VRMDT) with Sentria XR company, which spins out company from the University of Manchester. Multidisciplinary team meeting is considered on of very important departmental facilitators that helps in increasing patient outcomes, and reducing medical errors by enhancing knowledge sharing practices among healthcare professionals. Most of them faced many challenges that hinder in performing those meeting, and the lack of time, and place one of them. Additionally, after Covid-19 pandemic, most of the meetings were took place online to protect their life’s. Therefore, Creating VRMDT will help them to overcome those challenges.  
          A VRMDT software can be used as an alternative tool for performing those kind of meeting with new experience, which is close to the reality. Therefore, I am inviting a group of healthcare professionals who are involved in MDT to participate in System Usability Study (SUS). 
Completing the survey will take approximately 5-10 min. You have the option of withdrawing from this study at any time, and your participation is completely voluntary.
To complete the survey, kindly just click on the following link:

              The questionnaire does not ask for any personal information from any of the participants. In addition, it has been approved by ethical community in the University of Manchester. In the survey, all responses will be kept anonymously. Please keep in mind that there are no right or wrong responses, and that your own opinion is what matters important to us.
Please do not hesitate to contact me if you have any questions or concerns regarding this project, you may send an email to the address below:

Maryam.almashmoum@Student.manchester.ac.uk

**Regards**
Maryam Almashmoum

**The consent form**:
If you would like to participate in this survey, please consent the following statements.

| 1. I am aware that my participation in this study is voluntary and that I will be free to withdraw at any time without giving a reason and without detriment to myself. |  |
| --- | --- |
| 1. I understand that any data collected may be included in anonymous form in publication or conference presentations. |  |
| 1. I am aware that, if it is pertinent to my participation in this research, data acquired during the study may be examined by personnel from The University of Manchester or regulatory authorities. I agree to let these people have access to my data. |  |
| 1. I understand that a fully anonymised dataset will be deposited in an open data repository. |  |
| 1. I agree to participate in this study |  |

**Survey Questions**

**Part1: Demographics profile of respondents**

Please select the most appropriate answer.

1. Which hospital do you work at?
   1. The Christie Hospital
   2. Kuwait Cancer Control Center (KCCC)
2. Which department do you work in?
   1. Chemotherapy unit.
   2. Brachytherapy & Molecular Radiotherapy Unit
   3. Endocrine Unit & PDT
   4. Estates Department
   5. Haematology
   6. Laboratory
   7. Medical imaging department
   8. Medical oncology
   9. Nursing
   10. Nuclear medicine department
   11. Palliative care
   12. Physical therapy
   13. Physics/Radiotherapy
   14. Pharmacy
   15. Radiation oncology
   16. Radiology department
   17. Radiotherapy unit
   18. Radio pharmacy
   19. Radio pharmacy
   20. Rehabilitation unit
   21. Surgical oncology
   22. Other Technical Services
   23. Other......................................................
3. What is your role?
   1. Administration/clerical
   2. Anaesthetic
   3. Diagnostic Radiographer
   4. Histopathology
   5. Laboratory scientists
   6. Nuclear medicine technologist
   7. Nurse
   8. Pharmacists
   9. Radiology nurses
   10. Specialist doctor (Consultants)
   11. Others ..............................................
4. What is your sex?
   1. Male
   2. Female
   3. Prefer not to say
   4. None of the above
5. What is your age group?
6. Under 20 years
7. 20—30 years.
8. 30-40 years.
9. 40-50 years.
10. 50-60 years.
11. Above 60 years.
12. What is your highest education level?
    1. Diploma
    2. First degree (Bachelor)
    3. Master’s degree
    4. Doctorate degree
    5. Other...............................................
13. How many year you have been in this hospital?
    1. Less than 10 years
    2. 10-20 years
    3. 20-30 years
    4. More than 30 years

**System Usability Scale**

**Instructions**: For each of the following statements, mark one box that best describes your reactions to the VRMDT software today.

| The questions | SD | D | NAD | A | SA |
| --- | --- | --- | --- | --- | --- |
| 1. I think that I would like to use this software frequently |  |  |  |  |  |
| 2. I found this software unnecessarily complex. |  |  |  |  |  |
| 3. I thought this software was easy to use. |  |  |  |  |  |
| 4. I think that I would need assistance to be able to use this software. |  |  |  |  |  |
| 5. I found the various functions in this software were well integrated (e.g. whiteboard, and DICOM images) |  |  |  |  |  |
| 6. I thought there was too much inconsistency in this software |  |  |  |  |  |
| 7. I would imagine that most professionals would learn to use this software for MDT meetings very quickly. |  |  |  |  |  |
| 8. I found this software very cumbersome/awkward to use. |  |  |  |  |  |
| 9. I felt very confident using this software. |  |  |  |  |  |
| 10.I needed to learn a lot of things before I could going with this software |  |  |  |  |  |

**The 12 heuristics evaluation (Sutcliffe & Gault, 2004):**

| The heuristic items | No problem | Cosmic  Problem | Minor  problem | Major problem | Catastrophic |
| --- | --- | --- | --- | --- | --- |
| **1. Natural engagement. Interaction should approach the user’s expectation of interaction in the real world as far as possible** |  |  |  |  |  |
| 2. Compatibility with the user’s task and domain. The VE and behaviour of objects should correspond as closely as possible to the user’s expectation of real world objects; their behaviour; and affordances for task action. |  |  |  |  |  |
| 3. Natural expression of action. The representation of the self/presence in the VE should allow the user to act and explore in a natural manner and not restrict normal physical actions. |  |  |  |  |  |
| 4. Close coordination of action and representation. The representation of the self/presence and behaviour manifest in the VE should be faithful to the user’s actions. |  |  |  |  |  |
| 5. Realistic feedback. The effect of the user’s actions on virtual world objects should be immediately visible and conform to the laws of physics and the user’s perceptual expectations. |  |  |  |  |  |
| 6. Faithful viewpoints. The visual representation of the virtual world should map to the user’s normal perception, and the viewpoint change by head movement should be rendered without delay. |  |  |  |  |  |
| 7. Navigation and orientation support. The users should always be able to find where they are in the VE and return to known, present positions |  |  |  |  |  |
| 8. Clear entry and exit points. The means of entering and exiting from a virtual world should be clearly communicated. |  |  |  |  |  |
| 9. Consistent departures. When design compromises are used they should be consistent and clearly marked, e.g. cross-modal substitution and power actions for navigation. |  |  |  |  |  |
| 10.Support for learning. Active objects should be cued and if necessary explain themselves to promote learning of VEs. |  |  |  |  |  |
| 11.Clear turn-taking. Where system initiative is used it should be clearly signalled and conventions established for turn-taking. |  |  |  |  |  |
| 12.Sense of presence. The user’s perception of engagement and being in a ‘real’ world should be as natural as possible. |  |  |  |  |  |

Add any comments regarding the software
